# Metabolic Reconfiguration Activates Stemness and Immunomodulation of PDLSCs

**DOI:** 10.3390/ijms23074038

**Published:** 2022-04-06

**Authors:** Payal Arora, Wen Li, Xiaobin Huang, Wenjing Yu, Ranran Huang, Qian Jiang, Chider Chen

**Affiliations:** 1 Early-Research Oral Care, Colgate-Palmolive Company, Piscataway, NJ 08854, USA; pmvarora@gmail.com; 2 Department of Oral & Maxillofacial Surgery & Pharmacology, School of Dental Medicine, University of Pennsylvania, Philadelphia, PA 19104, USA; ewen74@163.com (W.L.); bmwzzhsh@upenn.edu (X.H.); wenjingy@upenn.edu (W.Y.); huangrr8@163.com (R.H.); njuqianqian@foxmail.com (Q.J.); 3 Center of Innovation & Precision Dentistry, School of Dental Medicine, School of Engineering and Applied Sciences, University of Pennsylvania, Philadelphia, PA 19104, USA

**Keywords:** metabolic reconfiguration, immunomodulation, periodontal ligament derived stem cells (PDLSCs), mesenchymal stem cells (MSCs), RNA-sequencing (RNA-seq), prostaglandin E2 (PGE2), Indoleamine 2,3 dioxygenase (IDO)

## Abstract

Periodontal ligament derived stem cells (PDLSC) are adult multipotent mesenchymal-like stem cells (MSCs) that can induce a promising immunomodulation to interact with immune cells for disease treatment. Metabolic reconfiguration has been shown to be involved in the immunomodulatory activity of MSCs. However, the underlying mechanisms are largely unknown, and it remains a challenging to establish a therapeutic avenue to enhance immunomodulation of endogenous stem cells for disease management. In the present study, RNA-sequencing (RNA-seq) analysis explores that curcumin significantly promotes PDLSC function through activation of MSC-related markers and metabolic pathways. In vitro stem cell characterization further confirms that self-renewal and multipotent differentiation capabilities are largely elevated in curcumin treated PDLSCs. Mechanistically, RNA-seq reveals that curcumin activates ERK and mTOR cascades through upregulating growth factor pathways for metabolic reconfiguration toward glycolysis. Interestingly, PDLSCs immunomodulation is significantly increased after curcumin treatment through activation of prostaglandin E2-Indoleamine 2,3 dioxygenase (PGE2-IDO) signaling, whereas inhibition of glycolysis activity by 2-deoxyglucose (2-DG) largely blocked immunomodulatory capacity of PDLSCs. Taken together, this study provides a novel pharmacological approach to activate endogenous stem cells through metabolic reprogramming for immunomodulation and tissue regeneration.

## 1. Introduction

Stem cell activity in the adult phase is shaped by external stimulations and internal processes, which contribute to tissue homeostasis and regeneration [1]. Mesenchymal stem-like cells (MSCs) are adult stem cells with the capacity of self-renewal and multipotential differentiation into different cell lineages, including osteoblasts, adipocytes, chondrocytes, and neuronal cells [2]. Systemic infusion of MSCs can interact with a variety of innate and adaptive immune cells, such as lymphocytes, macrophages, and neutrophils to regulate immune responses and ameliorate immune-related disorders such as graft versus host disease [3], systemic lupus erythematosus [4], and systemic sclerosis [5]. As MSCs extensive distribution in adult tissues/organs, these unique properties make MSCs an ideal cell source for regenerative medicine [2,6]. MSCs isolated from craniofacial tissues, such as periodontal ligament derived stem cells (PDLSCs) exhibit powerful abilities for orofacial repair and regeneration because of their commitment toward craniofacial tissues [7]. Despite of the current progress in MSC-based therapy, the therapeutic outcome is not consistent due to the rapid apoptosis of transplanted MSCs caused by unfavored host microenvironment and lack of understanding in detail molecular mechanisms [8]. Therefore, autotherapies through activation of somatic stem cells have become the new paradigm for regenerative medicine [9].

Dynamic metabolic conversion sustains tissue and stem cell properties [10]. The bivalent energetic status, including glycolysis and mitochondrial respiration, balances stem cell fate selection, which supports cell survival and growth and regulates MSC immunomodulation through generating specific cytokine secretory profiles to govern immune regulation [11,12,13,14]. As metabolic status fine-tunes several aspects of cellular behaviors, dissecting the dynamic metabolic configuration that governs MSC fate to adapt specific microenvironment and to manipulate their immunomodulation is critical for developing novel strategies to enhance MSC-based therapy in treating tissue degenerative and immune-related disorders.

Curcumin is a natural lipophilic compound that displays abilities to inhibit inflammatory conditions and enhance tissue regeneration in several tissues and organs [15,16]. Recently, tetrahydrocurcumin (T-curcumin), a major natural curcumin metabolite, draws more attention in biomedical research because of its superior properties such as solubility in water, bioavailability, stability, and anti-inflammation ability. At the cellular level, curcumin maintains MSC homeostasis for obesity and osteoporosis through activating endogenous stem cell capabilities and improving the osteo-lineage commitment of MSCs [17]. However, the mechanisms of curcumin in MSC-based regenerative medicine and immunomodulation are largely unknown. In the present study, our data demonstrated that curcumin induces cell proliferation, elevates osteogenic and chondrogenic differentiation, and improves PDLSC immunomodulation through reprogramming metabolic status toward glycolysis. In summary, our findings provide a unique strategy of MSC-based regenerative medicine using a pharmacological natural compound.

## 2. Results

### 2.1. Transcriptomic Profiles of Curcumin Treated PDLSCs

**Figure 1:**
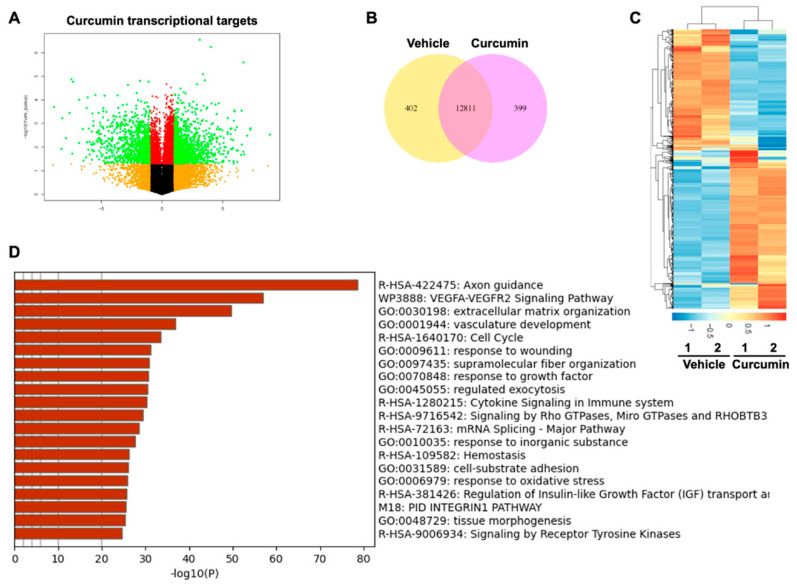
Transcriptome analysis of PDLSC with curcumin treatment. (**A**) Volcano plot showing the log2 fold changes of curcumin target gene expression with 5 μM curcumin treatment on the *x*-axis and the statistical *p*-value on the *y*-axis. (**B**) Venn diagram of differentially expressed genes in curcumin treated PDLSCs with *p* < 0.05. Vehicle: upregulated transcripts with vehicle treatment. Curcumin: upregulated transcripts with curcumin treatment. (**C**) Heat map of RNA-seq analysis data showing the differentially regulated genes with curcumin treatment. Gene expression is shown in normalized log2 counts per million. Differentially expressed genes were selected based on the fold change. (**D**) Enrichment analysis of Gene Ontology (GO) for differentially regulated curcumin target genes. Only the top 20 false discovery rate (FDR) enrichment of GO terms from “biological process” category were listed.

To explore the biological function of curcumin in PDLSCs, RNA-sequencing (RNA-seq) analysis was performed to profile transcriptomic levels with or without curcumin treatment. A total 801 transcripts were identified with significant changes of their expression, log2 fold change (FC) > 1 and FC < −1, and *p* value < 0.01, after curcumin treatment in PDLSCs when compared to the vehicle treated group (Figure 1A). Among these, 402 (50.2%) were down-regulated and 399 (49.8%) up-regulated upon curcumin treatment (Figure 1B). The fact that 50.2% of the targets became downregulated upon curcumin treatment suggests a potential role of curcumin contributing to transcriptional repression or preventing activation. Gene oncology (GO) enrichment analysis terms over the 801 curcumin target genes showed that the most enriched were related to growth factor and cell cycle signaling categories (Figure 1C,D).

### 2.2. Curcumin Promotes PDLSC Self-Renewal and Differentiation Capabilities

**Figure 2:**
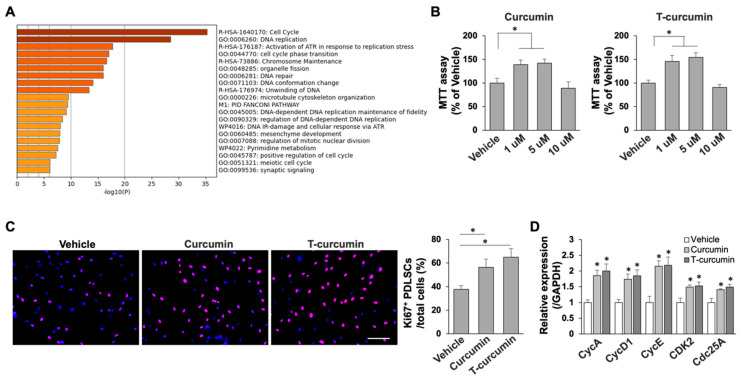
Curcumin elevated PDLSC proliferation. (**A**) Curcumin significantly activated cell cycle and cell proliferation pathways in PDLSCs by GO enrichment analysis. (**B**) MTT assay showed that curcumin treatment promoted PDLSC viability. (**C**) Immunofluorescence cellular staining by using Ki67 antibody showed that curcumin treatment greatly elevated Ki67^+^ cell percentage compared to vehicle treated group. Scale bar, 25 μm. (**D**) qPCR analysis showed that the levels of cell cycle genes were significantly elevated with curcumin treatment in PDLSCs. * *p* < 0.005. PDLSCs in passage 3 were used in all the assays. All experiments were at least repeated three times. Error bars represent the s.d. from the mean values.

Next, we asked whether the curcumin target genes contribute to the activation of PDLSC proliferation and differentiation capabilities. We then reanalyzed GO enrichment to focus on cell proliferation genes and found curcumin treatment significantly activated cell cycle related pathways, particularly cell cycle, DNA replication, chromosome maintenance, and DNA repair (Figure 2A). We then measured PDLSC cellular metabolic activity with curcumin treatment by MTT assay which has been widely used to assess cell viability. We examined two commercially available curcumin and T-curcumin and revealed that 1 and 5 μM curcumin treatment largely activated cell viability in PDLSCs (Figure 2B). Similar activated effect was found in T-curcumin treatment group. Next, immunofluorescence (IF) staining was performed with cell proliferation marker Ki67 antibody to show that curcumin treatment largely increased Ki67^+^ PDLSC percentage when compared to vehicle treatment group (Figure 2C). In order to confirm the upregulation of molecular targets in cell cycle after curcumin treatment, we performed quantitative PCR (qPCR) to determine that cell cycle genes cyclin A (CycA), cyclin D-1 (CycD1), cyclin E (CycE), cyclin-dependent kinase-2 (CDK2), and cell division cycle 25 A (Cdc25A) were greatly activated by curcumin treatment (Figure 2D), indicating curcumin promoted PDLSC proliferation through activation of cell cycle.

**Figure 3:**
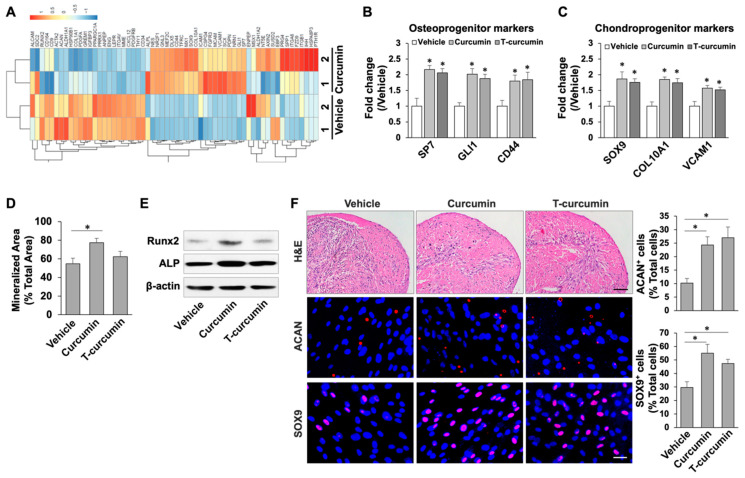
Curcumin increased the capabilities of osteogenic and chondrogenic differentiation in PDLSCs. (**A**) Heat map of RNA-seq analysis data showing the PDLSC function-related genes that were differentially regulated by curcumin treatment. (**B**,**C**) qPCR assay showed the significantly elevated levels of osteoprogenitor markers (**B**) and chondroprogenitor markers (**C**) in curcumin treated PDLSCs. (**D**) Alizarin red staining showed that curcumin treated PDLSCs has increased capacity to form mineralized nodules under osteoinductive conditions. (**E**) Western blot analysis showed the expression levels of the osteogenic genes RUNX2 and ALP were greatly increased in curcumin treated PDLSCs under osteoinductive conditions. β-actin was used as a loading control. (**F**) Histological analysis showed chondrogenic differentiation of vehicle and curcumin treated PDLSCs. Scale bar, 25 μm. IF staining showed increased ACAN^+^ and SOX9^+^ cells after curcumin treatment in PDLSCs. Scale bar, 25 μm. * *p* < 0.005. Error bars represent the s.d. from the mean values.

We then aimed to examine whether curcumin treatment could stimulate multilineage differentiation properties of PDLSCs. The RNA-seq analysis identified 60 MSC function-related genes that significantly changed their expression, log2 FC > 1 and FC < −1 and *p* value < 0.01, after curcumin treatment in PDLSCs. Among them, 24 (40%) were down-regulated and 36 (60%) up-regulated upon curcumin treatment (Figure 3A), indicating curcumin highly activated the MSC function of PDLSCs. We further confirmed these data by qPCR analysis to showed curcumin treatment significantly elevated osteoprogenitor markers including osterix (Osx/SP7), CD44 and GLI family zinc finger 1 (GLI1) (Figure 3B) and chondroprogenitor markers such as SRY-box transcription factor 9 (SOX9), collagen type X alpha 1 (COL10A1) and vascular cell adhesion molecule 1 (VCAM1) (Figure 3C). These data prompted us to examine the capabilities of osteogenesis and chondrogenesis in PDLSCs with curcumin treatment. When cultured under osteogenic stimulations, curcumin treated PDLSCs exhibited superior osteogenesis, as indicated by elevated mineralized nodule formation (Figure 3D) and levels of the osteogenic genes runt-related transcription factor 2 (RUNX2) and alkaline phosphatase (ALP), respectively (Figure 3E). In parallel, under chondrogenic inductive conditions, curcumin treated PDLSCs showed an increased capacity to differentiate into chondrocytes with the significantly elevated expression of aggrecan (ACAN) and SOX9 by immunostaining analysis (Figure 3F). Collectively, our findings indicated that curcumin promotes self-renewal and multipotent differentiation properties of PDLSCs.

### 2.3. Curcumin Regulates ERK and mTOR Cascades through Activation of Growth Factor Signaling for Metabolic Reconfiguration in PDLSCs

**Figure 4:**
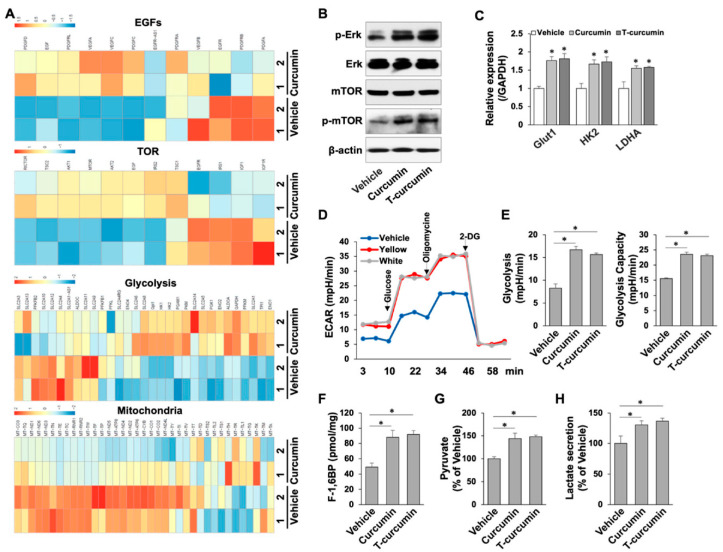
Curcumin reprogramed energy metabolism toward glycolysis through growth factor pathways and their downstream ERK and mTOR cascades. (**A**) Heat map of RNA-seq transcriptomic data showing the growth factor genes and Akt-mTOR cascades were differentially regulated by curcumin treatment in PDLSCs. In addition, energy metabolism was re-configurated toward glycolysis after curcumin treatment. (**B**) Western blot analysis showed the expression levels of p-ERK, ERK, mTOR, and p-mTOR in PDLSCs with or without curcumin treatment. (**C**) qPCR analysis confirmed that glycolytic genes, Glut1, HK2, and LDHA were significantly elevated after curcumin treatment in PDLSCs. (**D**) Seahorse assay results for ECAR of PDLSCs with or without curcumin treatment. (**E**) The contribution of associated parameters including glycolysis and glycolytic capacity to the total ECAR was plotted. (**F**) Intracellular concentrations of fructose-1,6-bisphosphate in PDLSCs with or without curcumin treatment. (**G**,**H**) Curcumin treated PDLSCs had increased glycolytic intermediates pyruvate (**G**) and lactate (**H**). * *p* < 0.005. Error bars represent the s.d. from the mean values.

Next, we aimed to explore the signaling targets of curcumin in PDLSCs. To further confirm our results in Figure 1D, we reanalyzed RNA-seq data with pathway enrichment to show that several growth factors, such as platelet-derived growth factor (PDGF), vascular endothelial growth factor (VEGF) and epidermal (EGF) pathways, were greatly activated in curcumin treated PDLSCs (Figure 4A). As one of the major downstream targets of growth factor pathways is ERK mitogen-activated protein kinase (MAPK) and mammalian target of rapamycin (mTOR) signaling [18], the analysis showed that curcumin treatment significantly activated ERK and mTOR signaling through phosphorylation of ERK (p-ERK) and mTOR (p-mTOR) by Western blot analysis in PDLSCs (Figure 4A,B). It has been shown that ERK and mTOR signaling regulate dynamic metabolic switches [19,20]. Our RNA-seq data further revealed that curcumin treatment was able to increase the metabolic reprogramming toward glycolysis from mitochondrial respiration in PDLSCs (Figure 4A). To further confirm the activation of glycolysis in curcumin treated PDLSCs, qPCR was performed to show glycolytic genes glucose transporter-1 (Glut1), hexokinase-2 (HK2), and lactate dehydrogenase A (LDHA) were highly activated in curcumin treated PDLSCs (Figure 4C). To further examine whether higher glycolytic metabolic protein expression levels in curcumin-treated PDLSCs can contribute to glycolysis, we performed a Seahorse assay using an XFe96 analyzer with live cells to measure simultaneously glycolytic activities. The elevated glycolytic activity was found in curcumin treated PDLSCs as demonstrated by elevated extracellular acidification rates (ECAR), glycolysis rates, and glycolysis capacity when compared to vehicle treated cells (Figure 4D,E), implying curcumin treated PDLSCs undergo metabolic reconfiguration through activation of glycolysis. Metabolite analysis also showed that curcumin treated PDLSCs had high levels of glucose metabolism intermediates, such as fructose 1,6-bisphosphate (F-1,6BP, Figure 4F), consistent with increased pyruvate (Figure 4G) and lactate (Figure 4H) amounts.

### 2.4. Curcumin Is Associated with PDLSC Immunomodulation through PGE2-IDO Pathway

**Figure 5:**
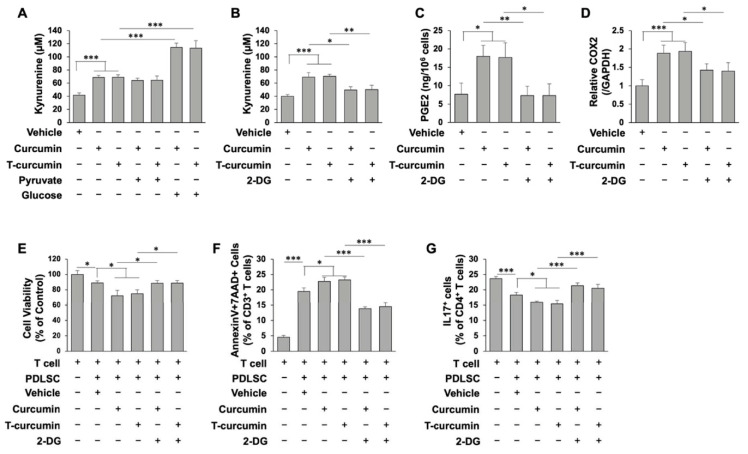
Glycolysis is required for PGE2/IDO secretion in PDLSC immunomodulation with curcumin treatment. (**A**) Production of kynurenine was significantly increased in curcumin treated PDLSCs. Addition of glucose, but not pyruvate, significantly increased kynurenine levels in curcumin treated PDLSCs. (**B**) IDO activity with or without glycolytic inhibitor, 2-DG treatment in the presence of curcumin in PDLSCs. (**C**) PGE2 level in cell culture supernatant with or without 2-DG treatment in the presence of curcumin in PDLSCs. (**D**) qPCR analysis of *COX2* mRNA level with or without 2-DG treatment in the presence of curcumin in PDLSCs. (**E**) Cell viability of activated T cells after co-cultured with PDLSCs with or without 2-DG treatment in the presence of curcumin. (**F**) PDLSCs induced annexinV^+^7AAD^+^ double positive apoptotic CD3^+^ T cells in a PDLSC/T-cell co-culture system in vitro. Curcumin treatment further increased annexinV^+^7AAD^+^ double positive apoptotic CD3^+^ T cells, while 2-DG largely blocked curcumin-induced immunomodulation of PDLSCs. (**G**) PDLSCs could inhibit CD4^+^IL17^+^ Th17 differentiation in vitro. Curcumin treatment further diminished Th17 differentiation, whereas 2-DG significantly reduce the inhibitory effect of curcumin. Error bars represent the s.d. from the mean values. *** *p* < 0.005; ** *p* < 0.01; and * *p* < 0.05.

Recently, immunomodulatory properties have been explored as an important characteristic of PDLSCs, by which systemic infusion of PDLSCs showed promising outcomes to treat a variety of immune disorders. We next examined whether curcumin is able to regulate PDLSC-mediated immunomodulation. To determine whether curcumin-mediated glycolysis activation is required for the elevation of immunosuppressive properties, PDLSCs were cultured with glucose or pyruvate in the absence or presence of curcumin. Kynurenine as a marker for the PDLSC immunomodulation was evaluated [21]. Our data revealed that curcumin treatment in the absence of glucose or pyruvate significantly increased kynurenine levels, which may be contributed by existing intracellular metabolites utilized for Indoleamine 2,3 dioxygenase (IDO) production. While 2 mM sodium pyruvate treatment alone did not increase kynurenine concentration compared with control groups, addition of glucose for 48 h significantly increased the level of kynurenine, indicating that glycolysis, but not pyruvate, metabolism is required for IDO production (Figure 5A). To further confirm the role of glycolysis in PDLSC immunomodulation, 2-deoxyglucose (2-DG) was used to inhibit glycolysis in the culture with 20 mM glucose to show the reduced kynurenine level (Figure 5B). The effect of 2-DG was also discovered in diminished prostaglandin E2 (PGE2) secretion and cyclooxygenase 2 (COX2) mRNA levels (Figure 5C,D). These findings indicated that glycolysis, but not pyruvate, metabolism provides the energy and carbon source for IDO and PGE2 production in PDLSC immunomodulation.

It has been shown that IDO plays a critical role in MSC-mediated T cell suppression [21]. In this regard, we assessed the functional role of curcumin-mediated high IDO production in the immunomodulatory properties of PDLSCs. Naïve T cells were activated by CD3 and CD28 antibodies for three days and then co-cultured with curcumin pre-treated PDLSCs containing 2-DG using a Transwell co-culture system. Curcumin pre-treated PDLSCs showed a marked inhibition of T cell viability when compared to vehicle treated PDLSCs (Figure 5E). 2-DG treatment largely blocked the inhibitory effects of curcumin in T cell viability (Figure 5E). Moreover, curcumin treatment showed a significantly elevated capacity to induce activated T cell apoptosis, whereas adding 2-DG in the culture, the number of apoptotic T cells was significantly reduced (Figure 5F). As Th17 has been shown to heavily involved in the pathogenesis of many autoimmune disorders, our data also showed that PDLSCs could inhibit Th17 differentiation with a more prominent effect observed with curcumin treatment (Figure 5G). Treatment of 2-DG largely abolished the inhibitions of Th17 differentiation (Figure 5G). In summary, our findings revealed that curcumin can enhance PDLSC-mediated T cell suppression through activation of glycolysis activity.

## 3. Discussion

MSC-based therapy provides a promising treatment outcome for tissue regeneration and disease amelioration, by which MSCs regulate tissue homeostasis and interplay with a variety of immune cells for immunomodulation [3,4,5,21]. Natural compounds (NC) are biologically active substances derived from natural products, including herbal extracts and woody plant-derived bioactive compounds. Recently, NC have been shown to regulate the immune response with low adverse side effects, which provides new approaches for immune modulation and can be promising therapeutic avenues in preventing chronic diseases [22,23]. Curcumin is a promising pharmacological target in regulating cell viability, cell proliferation, and anti-inflammatory properties, particularly in promoting osteogenesis of bone marrow derived MSCs [17,24]. In the present study, we showed that curcumin treatment significantly elevates stem cell function of PDLSCs through activation of osteogenic and chondrogenic markers, improvement of self-renewal and multi-lineage differentiation abilities, and up-regulation of immunomodulatory capacities. By a mechanistic study, our data reveal that curcumin-enhanced grow factor signaling activates downstream ERK and mTOR cascades, which may drive the expressions of downstream genes for osteogenesis and metabolic reconfiguration, respectively [5,13,25]. The experimental evidence in this study links curcumin to PDLSC-mediated immunomodulation and suggests a novel therapeutic avenue to activate endogenous adult stem cells for autotherapies.

Profiling the gene expression levels of stem cells under certain conditions by RNA-seq analysis is a promising and reliable approach to prioritize genetic variants [26]. In this study, RNA-seq analysis identifies that grow factor pathways, such as EGF and PDGF signaling, are the downstream targets of curcumin in PDLSCs. These growth factors through downstream ERK and mTOR cascades are well known as the key mediators in regulating MSC self-renewal and differentiation that play a key role for stem cell-mediated tissue regeneration [27]. In addition, our RNA-seq data links ERK/mTOR cascades to metabolic reconfiguration toward glycolysis, which plays a regulatory role in PDLSC immunomodulation [13,28]. These findings indicate that RNA-seq analysis provides the comprehensive transcriptomic profiles and related biological processes in curcumin treated PDLSCs.

The immunomodulatory properties of MSCs mediate proliferation, differentiation, and apoptosis of several major types of immune cells, by which systemic transplantation of MSCs or MSC-derived extracellular components yields promising therapeutic outcomes for a number of immune-related diseases [5,29,30]. Mechanistically, MSCs are able to interplay with immune cells through cell–cell interaction [31] or secretion of anti-inflammatory factors [32]. In this study, we revealed that curcumin improves PDLSC-mediated immunosuppression of T cells through activation of PGE2-IDO cascades which is a key immunosuppressive pathway [33] and regulated by curcumin-mediated glycolytic activation. Collectively, our experimental evidence explores an mTOR/glycolysis/PGE2-IDO cascades in the regulation of PDLSC immunomodulation to improve stem cell-based clinical therapies.

Dynamic metabolic conversion supports tissue- and life-stage-specific functions of stem cells [10,34]. Upon sensing extrinsic microenvironmental stimulations or intrinsic genetic/epigenetic regulations, cells reprogram their metabolic scheme to adapt functional demands [35]. However, the molecular reconfiguration regulating the MSC metabolic homeostasis for immunomodulation is still largely unknown. Our findings suggest that curcumin treatment largely induces anti-inflammatory capacities and activates PDLSC function through reprogramming PDLSC transcriptomic networks and metabolic status. The switch of bivalent energetics program between mitochondrial oxidative phosphorylation (OXPHOS) and anaerobic glycolysis controls adenosine triphosphate (ATP) generation for cell proliferation and differentiation [36]. Our RNA-seq analysis and Seahorse assay showed that curcumin treatment shifts metabolic profile toward glycolysis through ERK and mTOR cascades. As metabolic reprogramming of MSCs by pre-conditioning them using cytokines or metabolites significantly enhances their stem cell function and immunosuppressive potential [37,38], here we showed curcumin activates PDLSC immunomodulation to inhibits T cells through mTOR/glycolysis cascades, suggesting a pharmacological approach with limited exogenous intervention to achieve therapeutic effects. The new paradigm of regenerative medicine aims to promote somatic stem cell abilities to minimize invasive approaches for tissue regeneration [9]. Taken together, this translational study not only advances our knowledge in metabolic reconfiguration mediated PDLSC immunomodulation, but also provides a potential pharmacological approach for activation of endogenous adult stem cells for autotherapies.

## 4. Methods and Materials

Culture of PDLSCs. The human periodontal ligament (PDL) tissues were obtained from healthy patients undergoing third molar extractions as discarded biological samples from the University of Pennsylvania School of Dental Medicine following the approved institutional review board guidelines [7,39]. The freshly collected PDL tissues were digested with 2 mg/mL collagenase I (Worthington Biochem, Lakewood, CA, USA) and 4 mg/mL dispase (Sigma-Aldrich, St. Louis, MO, USA) for 1 h at 37 °C to obtain single-cell suspensions. For curcumin treatment, PDLSCs (0.2 × 10^6^/well) were seeded in 6-well culture plates under alpha minimum essential medium (α-MEM, Invitrogen, Waltham, MD, USA) supplemented with 15% fetal bovine serum (FBS, Atlanta Biologicals, Flowery Branch, GA, USA), 2 mM L-glutamine, 100 U/mL penicillin, and 100 μg/mL streptomycin (Invitrogen) with 5 μM curcumin for 24 h at 37 °C under 5% CO_2_ conditions. Dimethyl sulfoxide (DMSO), as curcumin solvent, treatment was used a vehicle control.

RNA isolation and Real-time qPCR assays. Total RNA was isolated using miRNeasy Mini Kit (Qiagen, Hilden, DE) according to the manufacturer’s instructions. High-Capacity cDNA Reverse Transcription kit (Applied Biosystems, Waltham, MS, USA) was used to prepare cDNA. qPCR assays were performed using SYBR Green qPCR Master Mix (Biomake, Houston, TX, USA) with gene specific primer pairs. All primers used in this study were synthesized and purified by Integrated DNA Technologies. The detail sequences of the primers are listed below. The gene expression was normalized to GAPDH. A CFX96 Real-Time PCR System (Bio-Rad, Hercules, San Diego, CA, USA) was used for qPCR analysis.

CycA-F: ACATGGATGAACTAGAGCAGGG; R: GAGTGTGCCGGTGTCTACTT
CycD1-F: GCTGCGAAGTGGAAACCATC; R: CCTCCTTCTGCACACATTTGAA
CycE-F: GGAGTTCTCGGCTCGCTCC; R: CGTCCTGTCGATTTTGGCC
CDK2-F: CCAGGAGTTACTTCTATGCCTGA; R: TTCATCCAGGGGAGGTACAAC
Cdc25A-F: GTGAAGGCGCTATTTGGCG; R: TGGTTGCTCATAATCACTGCC
Osx-F: TGCTTGAGGAGGAAGTTCAC; R: AGGTCACTGCCCACAGAGTA
GLI1-F: AGCGTGAGCCTGAATCTGTG; R: CAGCATGTACTGGGCTTTGAA
CD44-F: CTGCCGCTTTGCAGGTGTA; R: CATTGTGGGCAAGGTGCTATT
SOX9-F: AGCGAACGCACATCAAGAC; R: CTGTAGGCGATCTGTTGGGG
COL10A1-F: ATGCTGCCACAAATACCCTTT; R: GGTAGTGGGCCTTTTATGCCT
VCAM1-F: GGGAAGATGGTCGTGATCCTT; R: TCTGGGGTGGTCTCGATTTTA
Glut1-F: GGCCAAGAGTGTGCTAAAGAA; R: ACAGCGTTGATGCCAGACAG
HK2-F: GAGCCACCACTCACCCTACT; R: CCAGGCATTCGGCAATGTG
LDHA-F: TTGACCTACGTGGCTTGGAAG; R: GGTAACGGAATCGGGCTGAAT
COX2-F: CTGGCGCTCAGCCATACAG; R: CGCACTTATACTGGTCAAATCCC

RNA-seq analysis. After RNA extraction, mRNA was enriched using oligo(dT) beads. For library preparation, the cDNA was synthesized by using mRNA as template and random hexamers primer, followed by a custom second-strand synthesis and sequencing by Illumina sequencers (Illumina, San Diego, CA, USA). Reads obtained from RNA-seq were then aligned to the human reference genome. The differential expression between conditions was statistically assessed, and genes with FC > 1 and FC < −1 and *p* value < 0.01 were identified as differentially expressed. GO functional classification of differentially expressed genes was defined based on the QuickGo database. The enrichment of specific datasets was considered significant when the nominal *p* value was less than 0.05.

Antibodies and reagents. The antibody to ALP was purchased from Santa Cruz Biotechnology, Dallas, TX, USA. Antibodies to RUNX2, p-ERK, ERK, p-mTOR, mTOR, and Ki-67 were obtained from Cell Signaling Technology, Danvers, MA, USA. Anti-β-Actin antibody was purchased from Sigma-Aldrich. Anti-ACAN antibody was purchased from Abcam, Cambridge, UK, and anti-SOX9 antibody was purchased from Novus Biologicals, Littleton, CO, USA. Alexa Fluor 568 secondary antibody was purchased from Invitrogen.

Cell viability/proliferation assays. Human PDLSCs (1.0 × 10^4^/well) at passage 3–5 were seeded on 2-well chamber slides (Nunc, Waltham, MA, USA) and cultured to around 70% confluence with or without curcumin treatment. The cells were then fixed and incubated with anti-Ki67 antibody (1:200) for overnight at 4 °C, followed by 1 h secondary antibody staining. The number of Ki67-positive cells was indicated as a percentage of the total cell number. DAPI staining was used to count the total cell number. For MTT assay, PDLSCs were seeded on a 96-well culture plate (1 × 10^3^ cells/well). After a cell attached, curcumin or vehicle was applied for 12 h with regular MSC culture medium. After curcumin treatment, culture medium was replaced by serum-free medium, 10 μL MTT solution (Roche, Basel, Switzerland) was added into each well, incubated at 37 °C for 4 h, and then 150 μL DMSO was added into each well. The culture plate was placed in a rocking bed for 10 min at a low-speed oscillation. Finally, the optical density (OD) values were measured by a microplate reader at 490 nm. To avoid any background signal from the medium, the OD value of serum-free medium was subtracted for all the experimental samples.

In vitro osteogenic differentiation. For osteogenic differentiation assay in vitro, PDLSCs were cultured under osteogenic inductive conditions containing 180 mM KH_2_PO_4_ and 10^−4^ M dexamethasone sodium phosphate (Sigma) with or without curcumin treatment. Medium was replaced every 2–3 days. After 1 week of induction, cultured cells were harvested for osteogenic markers analysis by Western blot. For alizarin red staining, cells were induced for 4 weeks, followed by staining with alizarin red to assess mineralized nodule formation.

In vitro chondrogenic differentiation. Passaged cells were pelleted (1 × 10^6^ cells) in conical 15 mL polypropylene tubes, and the pellets were cultured overnight to separate the pellets from the tube. Human PDLSC pellets were then cultured under chondrogenic culture conditions containing 10^−4^ M dexamethasone sodium phosphate (Sigma) and 10 ng/mL human recombinant TGF-β1 (R&D Systems, Minneapolis, MN, USA) with or without curcumin treatment. Medium was replaced every 2–3 days, and cell pellets were harvested after 4-week induction. The pellets were fixed with 4% PFA for 4 h and sectioned for H&E and immunofluorescent staining.

Immunofluorescent staining. The histological sections were deparaffined by xylene and rehydrated by ETOH and ddH_2_O. After permeabilization with 0.1% Triton X-100 for 30 min, blocking buffer was used to reduce non-specific staining for 1h, and then the histological sections were incubated with primary antibodies (1:100) at 4 °C overnight, followed by 1 h secondary antibody staining (1:200) at room temperature. The slides were mounted and counterstained with DAPI (Vector Laboratories, Burlingame, CA USA).

Western blot analysis. Western blotting was performed as previously reported [5]. Briefly, total protein was extracted by M-PER mammalian protein extraction reagent (Thermo). Samples with 20 μg protein were applied and separated on the 4–12% NuPAGE gel (Invitrogen), followed by transferring to nitrocellulose membranes (Millipore, Burlington, VT, USA). Membranes were then blocked by blocking buffer (5% non-fat dry milk and 0.1% Tween-20) for 1 h and incubated with the primary antibodies (1:1000) diluted in blocking solution at 4 °C for overnight. After washing, HRP-conjugated secondary antibody (Santa Cruz Biotechnology; 1:10,000) was used to incubate with the membranes for 1 h at room temperature. Immunoreactive proteins were detected using SuperSignal West Pico Chemiluminescent Substrate (Thermo) and BioMax film (Kodak, Rochester, MI, USA). Anti-β-actin antibody was re-probed to quantify the number of loaded proteins after stripping.

Real-time measurement of cell metabolism. Extracellular acidification rate (ECAR) was measured using Seahorse XFe96 (Agilent Technologies, Santa Clara, CA, USA). Human PDLSCs (2.0 × 10^4^/well) were plated and cultured on Seahorse 96-well plates 24 h before experiment. ECAR measurement was performed over time following injection of 10 mM D-glucose, 1.5 μM oligomycin, and 20 mM 2-DG. Oxygen consumption was blocked by oligomycin, an ATP synthase inhibitor, whereas 2-DG, a glucose analog, was used to terminate glycolysis through competitive inhibition of glycolytic enzymes.

Metabolite analysis. Intracellular fructose-1,6-bisphosphate and pyruvate were measured using PicoProbe fructose-1,6-bisphosphate assay kit and pyruvate fluorogenic assay kit (BioVision, Waltham, MA, USA), according to the manufacturer’s instructions, respectively. The values of average intensity were normalized for protein content obtained, and then calculated as percentage to the control group. For lactate secretion, the medium contained lactate was measured using a lactate fluorogenic assay kit (BioVision). The values of average fluorescence intensity were normalized to cell number that was counted by hemocytometer, and then calculated as percentage to the control group.

IDO activity and PGE2 measurement. IDO enzyme activity was assessed by measuring Kynurenine level in the culture supernatant. Briefly, 200 μL cell culture supernatants were clarified by mixing with 30% trichloroacetic acid (TCA; Sigma) at 50 °C for 30 min, followed by centrifugation at 10,000× *g* for 10 min. An equal volume of Ehrlich’s reagent (2% 4-(Dimethylamino) benzaldehyde in glacial acetic acid) was added to the clarified supernatants, and optical density at 490 nm was measured. For secreted PGE2 measurement, clarified cell culture supernatants were quantified using a PGE2 parameter assay kit (R&D systems), according to the manufacturer’s instructions. The optical density at 450 nm was measured and normalized to cell number.

T-lymphocyte viability and apoptosis assay. The trans-well system (Corning, Corning, NY, USA) was used for co-culture experiments. Human PDLSCs (0.2 × 10^6^) were seeded on each lower chamber, pre-treated with curcumin with or without 2_DG for 24 h, and replaced the treatments to regular medium before co-culture. T-lymphocytes (1 × 10^6^) from spleen were pre-stimulated with plate-bound anti-CD3 (3 μg/mL) and soluble anti-CD28 (2 μg/mL) antibodies for 2 days in Dulbecco’s Modified Eagle’s Medium (DMEM, Lonza, Basel, Switzerland) with 10% heat-inactivated FBS, 50 μM 2-mercaptoethanol, 10 mM HEPES, 1 mM sodium pyruvate (Sigma), 1% non-essential amino acid (Cambrex, East Rutherford, NJ, USA), 2 mM L-glutamine, 100 U/mL penicillin and 100 mg/mL streptomycin. After activation, T cells were loaded in the upper chambers and cocultured for 2 days. To measure the T cell viability, cell counting kit-8 (MilliporeSigma) was used. Apoptotic T cells were detected by AnnexinV Apoptosis Detection Kit I (BD Bioscience, Franklin Lakes, NJ, USA) and then analyzed by FACS^Calibur^ flow cytometer with CellQuest software.

In vitro Th17 induction. CD4^+^CD25^−^ T-lymphocytes (1 × 10^6^/well), collected by using a CD4^+^CD25^+^ Treg isolation kit (Miltenyi Biotec, Bergisch Gladbach, Germany), were pre-stimulated with plate-bound anti-CD3 (3 μg/mL) and soluble anti-CD28 (2 μg/mL) antibodies for 2 days. Human PDLSCs (0.2 × 10^6^) were seeded on each well containing curcumin with or without 2_DG treatment for 24 h before co-culture. After replaced the culture to regular medium, the activated T cells were loaded on PDLSCs with recombinant TGFβ (2 ng/mL; R&D Systems) and interleukin-6 (50 ng/mL; Biolegend, San Diego, CA, USA). After 3 days, cells in suspension were collected and stained with anti-CD4-PerCP and anti-IL17-PE antibodies (Biolegend). Cells were analyzed by FACS^Calibur^ flow cytometer with CellQuest software.

Statistical analysis. To determine the number of samples for the described experiments, power analysis has been conducted based on the “Resource Equation” method because the effect size was unknown. To compare between two groups, independent unpaired two-tailed Student’s *t*-tests were used. For comparisons between more than two groups, one-way ANOVA analysis with the Bonferroni adjustment was performed. *p* values less than 0.05 were considered as statistically significant.

## Data Availability

Not applicable.
